# Ultrasensitive Multimodal Tactile Sensors with Skin‐Inspired Microstructures through Localized Ferroelectric Polarization

**DOI:** 10.1002/advs.202105423

**Published:** 2022-01-24

**Authors:** Young‐Eun Shin, Yong‐Jin Park, Sujoy Kumar Ghosh, Youngoh Lee, Jonghwa Park, Hyunhyub Ko

**Affiliations:** ^1^ School of Energy and Chemical Engineering Ulsan National Institute of Science and Technology (UNIST) 50 UNIST‐gil Ulsan 44919 Republic of Korea

**Keywords:** healthcare, interlocked microstructure, multifunctional sensor, self‐powered sensor, skin‐inspired tactile sensor, temperature sensor

## Abstract

Multifunctional electronic skins have attracted considerable attention for soft electronics including humanoid robots, wearable devices, and health monitoring systems. Simultaneous detection of multiple stimuli in a single self‐powered device is desired to simplify artificial somatosensory systems. Here, inspired by the structure and function of human skin, an ultrasensitive self‐powered multimodal sensor is demonstrated based on an interlocked ferroelectric copolymer microstructure. The triboelectric and pyroelectric effects of ferroelectric microstructures enable the simultaneous detection of mechanical and thermal stimuli in a spacer‐free single device, overcoming the drawbacks of conventional devices, including complex fabrication, structural complexity, and high‐power consumption. Furthermore, the interlocked microstructure induces electric field localization during ferroelectric polarization, leading to enhanced output performance. The multimodal tactile sensor provides ultrasensitive pressure and temperature detection capability (2.2 V kPa^−1^, 0.27 nA °C^−1^) over a broad range (0.1–98 kPa, −20 °C < Δ*T* < 30 °C). Furthermore, multiple simultaneous stimuli can be distinguished based on different response times of triboelectric and pyroelectric effects. The remarkable performance of this sensor enables real‐time monitoring of pulse pressure, acoustic wave detection, surface texture analysis, and profiling of multiple stimuli.

## Graphical abstract

Inspired by the structure and function of human skin, an ultrasensitive self‐powered multimodal sensor is demonstrated based on an interlocked ferroelectric copolymer microstructure. The triboelectric and pyroelectric effects of ferroelectric microstructures enable the simultaneous detection of mechanical and thermal stimuli in a single device, demonstrating potential applications in humanoid robots, wearable devices, and healthcare systems.

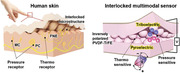

## 1 Introduction

Inspired by the somatosensory system of human skin composed of mechanoreceptors and thermoreceptors in the dermis, various types of multimodal electronic‐skins (E‐skins) have been developed to distinguish multiple stimuli,^[^ ^1^, ^2^, ^3^ ^]^ which is required for various applications, such as human–machine interfaces,^[^ ^4^ ^]^ artificial prosthetics,^[^ ^5^ ^]^ and healthcare monitoring.^[^ ^6^, ^7^ ^]^ In general, piezoresistive,^[^ ^8^ ^]^ capacitive,^[^ ^9^ ^]^ triboelectric,^[^ ^10^ ^]^ and piezoelectric^[^ ^11^ ^]^ sensors have been studied for the perception of mechanical stimuli, while thermoelectric,^[^ ^12^ ^]^ pyroelectric,^[^ ^13^ ^]^ and thermoresistive^[^ ^14^ ^]^ sensors have been studied for the perception of thermal stimuli.^[^ ^2^, ^15^ ^]^ In previous studies, multimodality has been achieved by integrating sensors with different working principles for different stimuli,^[^ ^16^, ^17^, ^18^, ^19^ ^]^ but this results in complicated processes, high manufacturing costs, and obtrusive structures. Hence, it is desirable to develop a multimodal E‐skin in a single sensing unit without signal interference. Li et al.^[^ ^20^ ^]^ presented dual‐mode sensors composed of a strain‐sensitive MXene–silver nanowire network and thermoelectric poly(3,4‐ethylenedioxythiophene):poly(styrenesulfonate) (PEDOT:PSS)–tellurium nanowire nanocomposite for the discrimination of mechanical and thermal stimuli. Han et al.^[^ ^21^ ^]^ described multimodal pressure/temperature/humidity sensors composed of ionic–electronic cellulose aerogels that decouple multiple signals based on the resistance change, electronic thermovoltage, and ionic thermovoltage. You et al.^[^ ^22^ ^]^ demonstrated an ionic‐conductor‐based multimodal E‐skin that can distinguish mechanical and thermal stimuli based on ion relaxation dynamics. Despite these remarkable advances, power consumption remains a critical issue for the development of portable and sustainable E‐skin. In this regard, self‐powered sensing mechanisms, such as triboelectricity, piezoelectricity, pyroelectricity, and thermoelectricity, have been proposed for multiple detection systems.^[^ ^10^, ^11^, ^12^, ^13^ ^]^ Although several studies have provided a decoupling strategy for multiple signals,^[^ ^23^, ^24^ ^]^ further study on the interpretation of multiparameters under simultaneous mechanical and thermal stimuli is required to enhance the sensing performance of multimodal sensing systems without signal interference.

Poly(vinylidene fluoride‐trifluoroethylene) (P(VDF‐TrFE)), a representative ferroelectric polymer, has been widely studied for self‐powered multimodal sensors owing to its outstanding piezoelectric/pyroelectric response and strong electronegativity in the triboelectric series. Several previous studies have boosted the pressure‐ and temperature‐responsive output performances of P(VDF‐TrFE) devices by ferroelectric polarization under an external electric field (E‐field), which is attributed to dipole alignment in ferroelectric polymers.^[^ ^24^, ^25^, ^26^, ^27^ ^]^ According to the “lightning rod effect,” the E‐field can be infinitely strengthened through the strong potential gradient caused by a rapid change in geometric curvature.^[^ ^28^, ^29^ ^]^ Furthermore, in insulator dielectrophoresis (iDEP), a spatially non‐uniform E‐field can be generated by embedding the microstructure of insulating obstacles in the channels, which enables manipulation of bioparticles.^[^ ^30^ ^]^ In this regard, the geometric manipulation of a ferroelectric copolymer strengthens the ferroelectric polarization through the localized E‐field. To the best of our knowledge, there are no reports on enhancing ferroelectric polarization with an intensive E‐field through microstructure engineering.

Here, we demonstrate a self‐powered multimodal E‐skin utilizing the interlocked microstructure of polarity‐modulated ferroelectric P(VDF‐TrFE). To mimic the stress concentration effect of the epidermal–dermal microstructure in human skin, we utilize an interlocked microstructure, which induces intensive ferroelectric polarization by localized E‐fields that can be concentrated in the area around the microstructures. This results in enhanced triboelectric output performance due to the increased surface charge density. Therefore, the high sensitivity and discriminability of multiple stimuli are achieved based on interlocked microridges. In addition, interlocked microridge structures can act as spacers to effectively vary the gap distance between opposing triboelectric surfaces. Consequently, by utilizing the enhanced ferroelectric polarization of P(VDF‐TrFE) microstructures, both pressure and temperature stimuli can be detected and discriminated based on the triboelectric and pyroelectric responses, respectively. Our devices provide high‐sensitivity pressure detection (2.2 V kPa^−1^) over a broad range of pressures (0.1–98 kPa), as well as high‐sensitivity temperature detection in the cooling (Δ*T* < 0) and heating (Δ*T* > 0) states (0.27 and 0.16 nA °C^−1^, respectively). Moreover, multiple signals from simultaneous pressure and temperature stimuli can be decoupled based on the electron flow direction and response time. The outstanding sensing performance, multimodality, simplicity, and self‐powered features of our E‐skin are highly desirable in healthcare sensors, human–machine interfaces, robotics, and artificial intelligence systems.

## 2 Results and Discussion

### 2.1 Design of Human Skin‐Inspired Ultrasensitive Tactile Sensors

**Figure 1:**
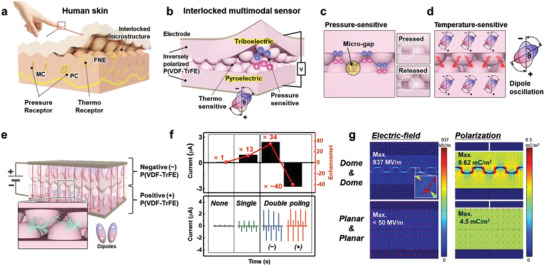
Skin‐inspired ultrasensitive multimodal sensors via enhanced ferroelectric polarization through an interlocked microstructure. a–d) Schematics of a) human skin and b) the interlocked multimodal sensor showing the functional characteristics with structural advantages for c) pressure and d) temperature sensing. e) Schematic of interlocked bilayer poling of the interlocked microstructure for intensive ferroelectric polarization. f) Dependence of the triboelectric output current on the polarization conditions. g) Finite element method (FEM) simulations of the localized E‐field distribution and polarization of the interlocked and planar structures.

**Figure** **1**a illustrates the sensory structure of human skin, including unique epidermal and dermal microstructures with sensory receptors. In particular, human skin perceives and distinguishes mechanical and thermal stimuli through tactile and temperature sensations, enabling the spatiotemporal recognition of the magnitude and location of touch and temperature stimuli. By mimicking this novel structure and multiple stimuli detection, we designed an interlocked microstructure with inversely polarized P(VDF‐TrFE) films, which simultaneously perform the role of pressure‐sensitive triboelectric and temperature‐sensitive pyroelectric layers (Figure 1b). This interlocked microstructure also causes E‐field localization during ferroelectric polarization, leading to highly improved sensing performance based on triboelectricity and pyroelectricity. In addition, for pressure detection, the interlocked microstructure causes extreme and transient changes in the contact area through effective stress localization at the small contact sites between the microridges, leading to an instantaneous triboelectric response to pressure.^[^ ^31^ ^]^ Furthermore, inversely polarized P(VDF‐TrFE) films with an interlocked microstructure can be effectively charged by contact electrification without additional bulky spacers owing to the micro‐gap induced by the interlocked structure, leading to pressure‐sensitive performance with a simple and thin device structure (Figure 1c). For temperature detection, despite the micro‐gap between the upper and lower P(VDF‐TrFE) films, the applied thermal stimulus can be effectively transferred through the contact sites between the microridges, leading to temperature‐sensitive performance (Figure 1d).

Our multimodal sensor is driven by triboelectric and pyroelectric effects, which are highly dependent on the polarized state of the P(VDF‐TrFE) films. To achieve highly polarized P(VDF‐TrFE) films with highly aligned dipoles, we utilized interlocked microstructures to induce E‐field localization during ferroelectric polarization. According to the “lightning rod effect,” a tapered metal structure enables the E‐field to be infinitely strengthened through a geometric singularity.^[^ ^28^, ^29^ ^]^ In the field of iDEP, bioparticles can be manipulated through a spatially non‐uniform E‐field, which is created by embedding the microstructure of insulating obstacles in a channel or flow field.^[^ ^30^ ^]^ Similarly, the ferroelectric polarization was enhanced by manipulating the E‐field using the interlocked microstructure in this study. When applying an E‐field to align the dipoles (post‐electric poling) of the interlocked P(VDF‐TrFE) bilayer, the top and bottom P(VDF‐TrFE) layers are inversely polarized, resulting in positive and negative triboelectric materials (Figure 1e). We term this post‐electrical poling “interlocked bilayer poling” which is distinct from “monolayer poling” (Figure S1a, Supporting Information).

Figure 1f shows the dependence of the triboelectric output current on the post‐electrical poling conditions. The triboelectric output currents were measured through repeated contact–separation cycles between the P(VDF‐TrFE) monolayer and an Al electrode (Figure S1b, Supporting Information). Compared to the negligible outputs (70 nA) for the non‐poled P(VDF‐TrFE), the polarized P(VDF‐TrFE) with monolayer poling exhibited a 13 times higher output current (900 nA). In particular, the polarized P(VDF‐TrFE) films after interlocked bilayer poling exhibited 34 and −40 times higher output currents (2.39 and −2.78 µA) with signals in opposite directions, which were based on the direction of the applied voltage. This result is attributed to the more intensive ferroelectric polarization through interlocked bilayer poling compared to that of non‐poling and monolayer poling due to the localized E‐field at the interface in the interlocked microstructure. To confirm this result, we simulated the resultant E‐field distribution and polarization of the interlocked microstructure and a planar structure at 1.4 kV using the finite element method (FEM) with COMSOL Multiphysics (Figure 1g). In this simulation, the film thickness was 15 µm, and the microstructure had a diameter of 10 µm, pitch of 15 µm, and height of 6 µm, which matched the experimental conditions. The simulated E‐field and polarization of the interlocked microdome structure (937 MV m^−1^, 9.62 mC m^−2^) were considerably higher than those of the overlapped planar structure (<50 MV m^−1^, 4.5 mC m^−2^), indicating that the E‐field can be intensely localized at the interface of the interlocked microdome structure, resulting in drastically enhanced ferroelectric polarization of P(VDF‐TrFE).

We further explored the optimized microstructures during interlocked bilayer poling. In iDEP, the geometry of the insulating obstacles affects the localization of the E‐field.^[^ ^30^, ^32^ ^]^ To investigate the effects of microstructure shape on the localized E‐field during interlocked bilayer poling, differently shaped microstructures (i.e., planar, dome, pyramid, and pillar) were fabricated using the micromolding process. The fabricated microstructures had equal feature sizes (diameter of 10 µm, pitch of 15 µm, and height of 6 µm), as observed in the scanning electron microscopy (SEM) images (Figure S2a). Figure S2b and c shows dependence of the simulated E‐field distribution and polarization on the shape of the microstructure. In particular, remarkable localization was observed for the microdome and micropyramid structures due to the rapid change in the radius of curvature in the microstructure.^[^ ^32^ ^]^ The microdome structure exhibited superior pressure‐sensitive triboelectric performance compared to that of the others (Figure S2d, Supporting Information), which is attributed to effective stress confinement and a large change in contact area as well as the localized E‐field.^[^ ^31^ ^]^ Compared to that of the non‐poled device, the microdome structure exhibited an 18 times higher output current, which is the highest output enhancement among the differently shaped microstructures (Figure S2e, Supporting Information). In addition, we investigated whether different compositions of overlapped structures (i.e., planar–planar, dome–planar, and dome–dome) affect E‐field localization during interlocked bilayer poling, which influences the triboelectric output performance (Figure S3a, Supporting Information). Figure S3b,c, Supporting Information show the dependence of the simulated E‐field distribution and polarization on the composition of the overlapped structures, which confirms that the interlocked dome–dome structure exhibits superior performance. Compared to that of the non‐poled device, the interlocked microdome structure exhibited an 18 times higher output current, which is the highest output enhancement among the different compositions of overlapped structures (Figure S3d,e, Supporting Information). Furthermore, to investigate the effect of the size of the microstructure, microdome structures with different diameters (i.e., 10, 20, and 40 µm) were fabricated (Figure S4a, Supporting Information). The microdome structure with the smallest diameter (10 µm) exhibited the highest output enhancement after electrical poling (Figure S4b,c, Supporting Information), which is attributed to the enhanced triboelectricity due to the increased localized E‐field affected by the microstructure geometry.^[^ ^33^ ^]^ Overall, the theoretical and experimental results match well, which indicates that the shape, size, and composition of the interlocked microstructure significantly affect the localized E‐field, polarization, and thus the triboelectric performance. Consequently, the ultrasensitive tactile sensor was optimized based on the interlocked microdome (10 µm) structure for intensive ferroelectric polarization and highly enhanced triboelectricity, which is attributed to E‐field localization, stress confinement, and a large change in the contact area.

### 2.2 Pressure and Temperature Detection with the Inversely Polarized and Interlocked P(VDF‐TrFE) Sensors

**Figure 2:**
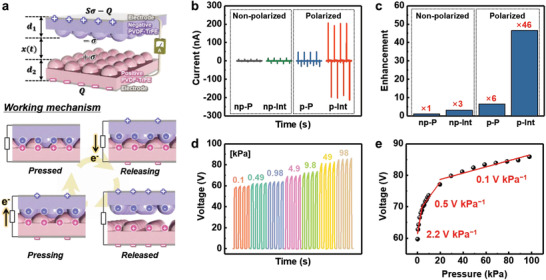
Triboelectric working mechanism and performances of sensors comprising inversely polarized P(VDF‐TrFE) with an interlocked microstructure. a) Schematic of triboelectric charge generation and the electron flow mechanism for interlocked P(VDF‐TrFE) microstructures with opposite polarization. b) Pressure‐sensitive triboelectric outputs for different types of sensors: non‐polarized/planar (np‐P), non‐polarized/interlocked (np‐Int), polarized/planar (p‐P), and polarized/interlocked (p‐Int). c) Output enhancement from each sensor compared to that of np‐P. d) Pressure‐dependent triboelectric output and e) linear fit of the variation in the triboelectric voltage with applied pressure (0.1–98 kPa).

**Figure** **2** shows the working mechanism and the pressure‐ and temperature‐sensing output performance of the sensor composed of inversely polarized interlocked P(VDF‐TrFE) microdome films as negative and positive triboelectric materials. The pressure sensitivity of the sensor originates from the conduction produced by contact electrification and electrostatic induction during repeated contact–separation cycles (Figure 2a). When the applied force reduces the micro‐gap distance between the interlocked structure, triboelectric surface charges are generated at the interface between the top and bottom P(VDF‐TrFE) films with opposite polarizations. During the restoration of the micro‐gap when the force is released, electrons flow through the external circuit until the accumulated charges reach equilibrium. When the two films approach each other again by the collapsed micro‐gap, electrons flow inversely to balance the charge. Consequently, continuous alternating triboelectric signals appear in response to repeated contact–separation movements. Figure S5, Supporting Information shows dominant triboelectric signals compared to piezoelectric ones from the planar structure of as‐poled P(VDF‐TrFE) films according to the existence of separation under the same force with 98 kPa, elucidating that the pressure‐dependent signals mainly come from the triboelectricity rather than piezoelectricity. Figure 2b,c and Figure S6, Supporting Information show the pressure‐sensitive triboelectric performances of different types of sensors: Non‐polarized/planar (np‐P), non‐polarized/interlocked (np‐Int), polarized/planar (p‐P), and polarized/interlocked (p‐Int). Compared to the subtle output (4.4 nA) for np‐P, p‐Int exhibited a 46 times higher output current (202 nA), which can be attributed to two factors: The opposite triboelectric polarity of the P(VDF‐TrFE) films in the contact pair and the effective contact–separation by the micro‐gaps between the two films due to the interlocked microstructure. Figure 2d,e shows the real‐time pressure detection of triboelectric sensors with polarized and interlocked P(VDF‐TrFE) films under a broad range of pressures (0.1–98 kPa, sensor size of 10 × 10 mm^2^). In the low‐pressure (<3.92 kPa), moderate‐pressure (3.92–19.6 kPa), and high‐pressure (19.6–98 kPa) regions, our sensors exhibited three different sensitivities (2.2, 0.5, and 0.1 V kPa^−1^, respectively). Figure S7, Supporting Information and Table S1, Supporting Information show the superiority of our sensor in terms of pressure sensing performance as well as device thickness, indicating the high performance with a simple device structure without additional spacer.

**Figure 3:**
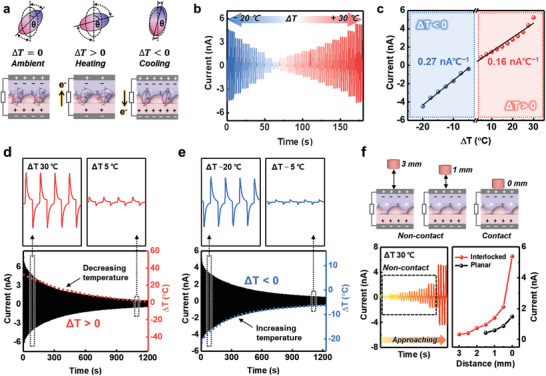
Pyroelectric working mechanism and performances of sensors with an interlocked P(VDF‐TrFE) microstructure. a) Schematic of pyroelectricity generation and the electron flow mechanism of the sensor with an interlocked P(VDF‐TrFE) microstructure upon different thermal stimuli. b) Temperature‐dependent pyroelectric output and e) linear fit of the variation in the pyroelectric current with the applied temperature (Δ*T*) from −20 to +30 °C. d,e) Highly responsive pyroelectric output under gradually varying thermal stimuli (Δ*T*); temperature d) decreasing from 30 to 5 °C and e) increasing from −20 to −5 °C. f) Pyroelectric performance as a proximity sensor with different distances between the sensor and thermal source (Δ*T* = 30 °C).

Moreover, our sensors with polarized P(VDF‐TrFE) films exhibit temperature‐sensitive pyroelectric performance through spontaneous changes in the polarization with the applied thermal stimulus (**Figure** **3**a). When the temperature is higher than the ambient state (Δ*T* > 0), a lower level of spontaneous polarization is induced (Δ*P*_s_ < 0) because the electric dipoles in P(VDF‐TrFE) oscillate further from their aligned axis compared to that in the ambient state (Δ*T* = 0). Accordingly, electrons flow through the external circuit because the quantity of induced charges in the electrodes is reduced. When the temperature is lower than the ambient state (Δ*T* < 0), a higher level of spontaneous polarization (Δ*P_s_* > 0) is caused by reduced dipole oscillation. Accordingly, electrons flow through the external circuit because the quantity of induced charges in the electrodes is increased. Consequently, repeated temperature change cycles induce continuously alternating pyroelectric current signals. Because the thermal conductivity of air is smaller than that of the P(VDF‐TrFE) film,^[^ ^34^ ^]^ the air gap between the two ferroelectric films hinders thermal transfer from the heat source to the sensors. Hence, the large gap in conventional triboelectric devices is not appropriate for prompt pyroelectric performance, although this large gap allows the triboelectric pair to be effectively separated during repeated contact–separation cycles to generate triboelectricity. Our sensors with an interlocked microstructure provide a remarkable compromise between triboelectric and pyroelectric performances as multimodal sensors. Figure S8 shows the pyroelectric performances of different types of temperature‐sensitive devices based on inversely polarized P(VDF‐TrFE) films: i) interlocked structure without a spacer, ii) planar structure without a spacer, and iii) planar structure with a spacer (Figure S8a, Supporting Information). The i) interlocked and ii) planar structures without a spacer exhibited pyroelectric signals with a similar scale, while the iii) planar structure with spacer exhibited substantially lower pyroelectric outputs (Figure S8b,c, Supporting Information). This result demonstrates that the interlocked microstructures used in the study are effective not only for triboelectric but also for pyroelectric performance owing to the micro‐gaps and contact sites between the two microstructured P(VDF‐TrFE) films.

Figure 3b shows the real‐time temperature sensing of the interlocked pyroelectric sensors without a spacer under a broad range of temperature changes (Δ*T*) from −20 to +30 °C at an interval of 2.5 °C. The pyroelectric output signals were highly responsive and depended on Δ*T*. In particular, when an object with a gradually varying temperature was repeatedly applied and withdrawn from the sensor, highly responsive time‐dependent pyroelectric signals were observed, which well match the dotted graphs generated by gradually decreasing the temperature of a hot object (Figure 3d) and increasing the temperature of a cold object (Figure 3e). Our pyroelectric sensors exhibited high sensitivities of 0.27 and 0.16 nA °C^−1^ in the cooling and heating states, respectively (Figure 3c). As shown in Figure 3f, our sensor with a high temperature sensitivity can also function as a proximity sensor. To characterize the proximity detection of our sensor, pyroelectric output signals were measured as a thermal source (Δ*T* = 30 °C) approached and withdrew repeatedly between an initial position (5 mm above the sensor) and specific position which was separated from the sensor with distances from 3 mm (non‐contact) to 0 mm (contact). Pyroelectric output signals are highly dependent on the distance between the thermal source and the sensor owing to heat transfer by thermal radiation from the thermal source.^[^ ^35^ ^]^ Because heat transfer is more effective through the P(VDF‐TrFE) film compared to the air gap, our sensor composed of an interlocked microstructure (8.8 µm) including a 1.9 µm micro gap exhibited remarkable proximity sensing performance compared to a control device composed of planar films with a large air gap (≈450 µm).

### 2.3 Effective Multimodal Sensing Performances of Simultaneous Pressure and Temperature Stimuli by Interlocked Microstructures

**Figure 4:**
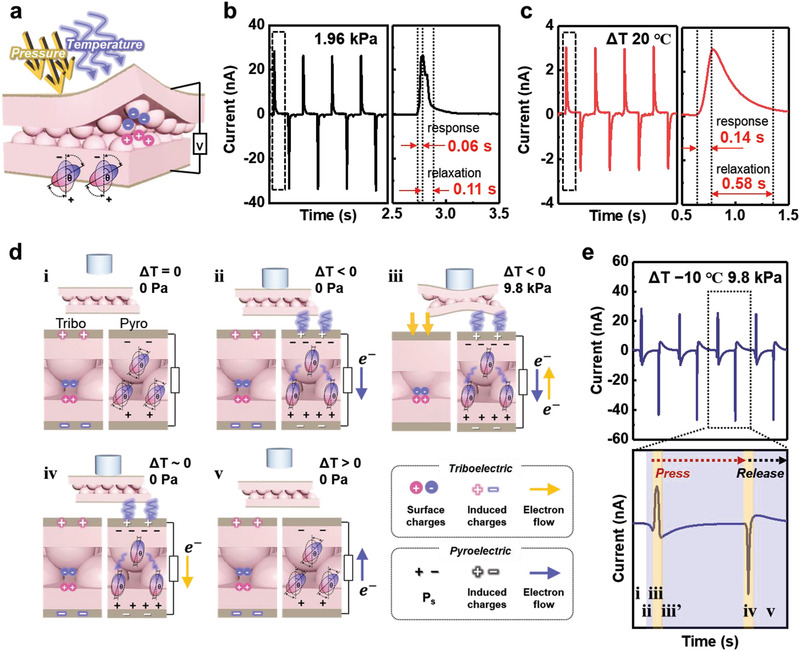
Multimodal sensing performance for the differentiation of simultaneous pressure and temperature stimuli in a single device. a) Schematic of the multimodal sensing of the sensor with an interlocked P(VDF‐TrFE) microstructure. b) Pressure‐responsive triboelectric output current upon only mechanical stimulus with a rapid response time. c) Temperature‐responsive pyroelectric output current upon only thermal stimulus with a slow response time. d) Working mechanism for bimodal signals in a single unit with triboelectric and pyroelectric effects. e) Multimodal signals and magnified peaks generated in response to multiple simultaneous stimuli (Δ*T* of −10 °C and 9.8 kPa).

The simultaneous triboelectric and pyroelectric performances allow our sensors with an interlocked microstructure to detect multiple stimuli (**Figure** **4**a). As discussed earlier, the interlocked microstructure of our sensors is suitable for both triboelectric and pyroelectric responses owing to the micro‐gap and contact sites between the P(VDF‐TrFE) microridges. Figure S9a, Supporting Information shows that the interlocked microstructure induces coupled triboelectric and pyroelectric signals under multiple stimuli, whereas the planar structure induces only pyroelectric signals without a noticeable triboelectric signal (Figure S9b, Supporting Information). As shown in Figure S10, Supporting Information and Table S2, Supporting Information, our sensor with an interlocked microstructure exhibits noticeable sensitivities for both pressure and temperature detection in comparison with those of previously reported multimodal sensors, indicating that the device in this study is superior in terms of multimodality. In particular, the response and relaxation times of the triboelectric output signals were 0.06 and 0.11 s under a mechanical stimulus of 1.96 kPa (Figure 4b). Furthermore, the pyroelectric response and relaxation times were 0.14 and 0.58 s under a thermal stimulus (Δ*T*) of 20 °C. The shorter response and relaxation times of the triboelectric peaks compared to those of the pyroelectric peaks enable simultaneous pressure and temperature stimuli to be distinguished by analyzing the coupled multiple signals, as discussed below.

Figure 4d shows the working mechanism for bimodal signals in a single unit corresponding to each state from two perspectives: the triboelectric and pyroelectric effects. When a cold object (Δ*T* = −10 °C) applies a periodic force of 9.8 kPa on the sensor, coupled signals composed of the opposite directional triboelectric and pyroelectric peaks appear (Figure 4e). The magnified output signal in Figure 4e is divided into three steps: i) the initial, ii–iii’) pressing, and iv–v) releasing states. Before any stimuli are applied (Figure 4d–i), there is no signal (Figure 4e‐i). When the object does not press but only contacts the sensor (Figure 4d‐ii), only a negative pyroelectric signal is caused by the increased spontaneous polarization (Δ*P*_s_ > 0) due to the negative temperature change (Δ*T* < 0) of the sensor without a pressure change (Figure 4e‐ii). Once a pressure (9.8 kPa) is applied on the sensor (Figure 4d‐iii), a positive sharp triboelectric signal is caused by the electron flow through the external circuit to maintain charge balance (Figure 4e‐iii). The response times to pressure and temperature stimuli are distinct: the positive triboelectric peak is sharp and fast (Figure 4e‐iii), while the negative pyroelectric peak is wide and slow (Figure 4e‐ii,iii’). When only the applied pressure is released but the object remains in contact with the sensor (Figure 4d‐iv), a negative triboelectric peak is induced by compensating charges because the micro‐gap between the interlocked microridges is restored (Figure 4e‐iv). Lastly, when the object is completely separated from the sensor (Figure 4d‐v), a positive pyroelectric peak is generated by the relatively decreased spontaneous polarization (Δ*P*_s_ < 0) due to the return of the sensor to room temperature (Figure 4e‐v). Based on the response and relaxation time of triboelectric and pyroelectric signals, the position of the pyroelectric peak (yellow arrow in Figure S11c, Supporting Information) overlapping with triboelectric peak can be expected.

**Figure 5:**
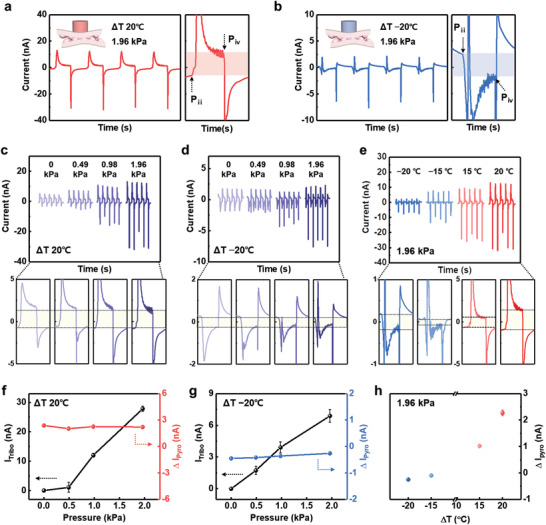
Multimodal output currents as a function of time and magnified peaks corresponding to simultaneous stimuli. a,b) Multiple signals under a) Δ*T* of 20 °C and 1.96 kPa and b) Δ*T* of −20 °C and 1.96 kPa. c,d) Multimodal output currents and magnified peaks with variations in pressure under Δ*T* of c) 20 °C and d) −20 °C. e) Multimodal output currents and magnified peaks with variations in temperature under 1.96 kPa. f,g) *I*_Tribo_ and Δ*I*_Pyro_ in (c) and (d), respectively. h) Δ*I*_Pyro_ in (e).

**Figure** **5** shows the multimodal response of our sensor under the simultaneously applied pressure and temperature stimuli. When applying the force (1.96 kPa) with a hot object (Δ*T* = 20 °C), sharp triboelectric and gradual pyroelectric peaks are coupled with the same direction, which is attributed to electron flow in the same direction from the triboelectric and pyroelectric effects. Furthermore, when the same force is applied with a cold object (Δ*T* = −20 °C), the peaks are coupled with opposite directions owing to the different electron flow directions from the triboelectric and pyroelectric effects. As shown in Figure 5a,b, the gradual pyroelectric peaks are cut by the following response peaks. Based on the multimodal working mechanism of the sensor (Figure 4d), *P*_ii_ and *P*_iv_ indicate points at which the pyroelectric peaks are cut by the following pyroelectric peak and triboelectric peak, respectively. In particular, a larger pyroelectricity has longer relaxation time before the equilibrium state is reached. This slow relaxation of the pyroelectric response can be utilized to differentiate the temperature response in the coupled signals by analyzing the pyroelectric current difference (Δ*I*_Pyro_) between P_ii_ and P_iv_. The temperature response can be calculated using the following equation:

(1) $$\Delta \;I_{\mathrm{Pyro}}=\;I_{P_{\mathrm{iv}}}-I_{P_{\mathrm{ii}}}$$

The information regarding the relative value and direction of Δ*T* can be obtained by Δ*I*_Pyro_ because Δ*I*_Pyro_ is directly proportional to Δ*T*, and a positive/negative Δ*I*_Pyro_ indicates a hot/cold thermal stimulus. Figure 5c,d show the pressure‐dependent output signals when Δ*T* is 20 and −20 °C, respectively. The triboelectric spike signals increase with increasing pressure, while Δ*I*_Pyro_ is constant at the same Δ*T* (Figure 5f,g). In particular, when the coupled triboelectric and pyroelectric peaks (Δ*T* > 0) have the same polarity direction, the triboelectric output current (*I*_Tribo_) is calculated using the following equation:

(2) $$I_{\mathrm{Tribo}\;(\Delta T\;>\;0)}=I_{\mathrm{Multiple}}\;-I_{\mathrm{Pyro}}$$

Here, *I*_Tribo (Δ_ *_T_*_> 0)_ is the pressure response of the coupled signals under Δ*T* > 0, and *I*_Pyro_ is the pyroelectric response at 0 Pa. As shown in Figure 5f, *I*_Tribo_ increases with increasing pressure, while Δ*I*_Pyro_ exhibits constant positive values under a fixed positive Δ*T* (20 °C). Similarly, Δ*I*_Pyro_ exhibits constant negative values under a fixed negative Δ*T* (−20 °C) (Figure 5g), wherein pressure‐responsive triboelectric peaks can be directly obtained without calculation because the signals can be decoupled owing to the opposite polarity directions of the triboelectric and pyroelectric peaks. In addition, when the temperature is variable with a fixed pressure (1.96 kPa), the temperature response (Δ*I*_Pyro_) depends on the absolute value of Δ*T* (Figure 5e,h). Therefore, our sensor can discriminate simultaneous temperature and pressure stimuli using Δ*I*_Pyro_ and *I*_Tribo_ induced by different responses and relaxations. This capability to distinguish multiple stimuli without external bias is advantageous for sustainable multimodal wearable devices (Table S2, Supporting Information).

### 2.4 Applications

**Figure 6:**
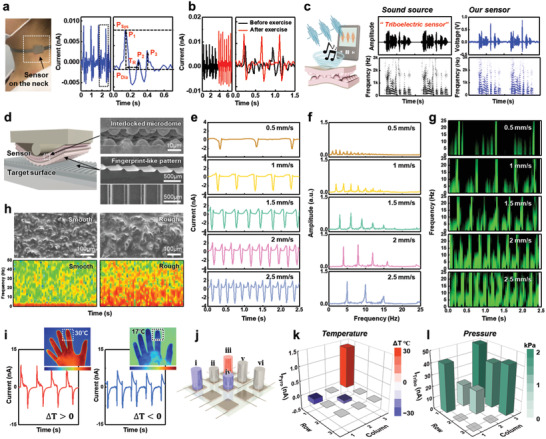
Applications of self‐powered multimodal sensors. a) Real‐time monitoring of the carotid pulse pressure and b) variation in the pulse pressure waves before and after physical exercise. c) Schematic of the acoustic sound wave detection and the corresponding time‐dependent sound waveforms and short‐time Fourier transform signals from a “triboelectric sensor” sound source and the sensor developed in this study. d) Schematic of the surface texture perception with SEM images of the sensor composed of an interlocked microstructure and fingerprint‐like PDMS pattern. e) Time‐dependent variation in triboelectric outputs under scanning of PDMS line pattern (periodic line pitch = 500 µm) at a different scanning speeds (0.5–2.5 mm s^−1^). f) FFT and g) STFT analyses of the triboelectric output signals at different scanning speeds. h) SEM images of sandpaper surfaces with different roughness values and the corresponding STFT analysis. i) Real‐time monitoring of finger touches; infrared images of hands and response signals corresponding to warm and cold fingers. j–l) Multimodal detection with a 3 × 3 device array. j) Schematic of the device with different objects and the corresponding k) temperature and l) pressure profiles based on the multimodal sensor: i) 2 kPa at Δ*T* −30 °C, ii) 1 kPa at room temperature, iii) 2 kPa at Δ*T* 30 °C, iv) 1 kPa at Δ*T* −30 °C, and v,vi) 2 kPa at room temperature.

The highly sensitive multimodal sensor developed in this study is advantageous for diverse wearable applications requiring self‐powered functions. In particular, the monitoring of blood pressure waveforms provides information for cardiovascular disease diagnosis in advance, which requires high sensitivity for a stable and exact output signal.^[^ ^36^, ^37^, ^38^ ^]^ The high pressure sensitivity of the sensor enables the monitoring of the weak pulse pressure of the carotid artery (**Figure** **6**a,b). The resulting pulse pressure waveform consisted of three distinct triboelectric peaks, which were induced from the main pulse pressure (*P*_1_) by contraction of the heart, and the reflected wave pressures (*P*_2_ and *P*_3_) from peripheral sites (Figure 6a).^[^ ^39^ ^]^ The indices of vascular health can be estimated by extracting the radial augmentation index (*AI_r_* = *P*_2_/*P*_1_) and round‐trip time (*T*_r_) of a reflected wave from the periphery of the hand based on these measured parameters, which are related to arterial stiffness according to the age of the individual. The average value of the augmentation index (*AI*_r_ = 0.27) is consistent with the literature data of healthy women in the same age group.^[^ ^40^ ^]^ Figure 6b shows the monitoring of pulse pressure before and after physical exercise. After physical exercise, the pulse rate increased from 83 beats per minute (BPM) to 133 BPM, and the signal intensity also increased, which is ascribed to the increased cardiac output to rapidly supply blood to the activated muscles. Our sensor is also promising for the detection of acoustic waves with a wide range of frequencies up to 3 kHz owing to its fast response time and remarkable pressure sensitivity (Figure 6c). To evaluate the acoustic sensing performance, a sound source (“triboelectric sensor”) was applied to the sensors using a commercial speaker. The waveforms from the original sound source (“triboelectric sensor” through a commercial speaker) were observed, and the corresponding spectrogram were converted by a short‐time Fourier transform (STFT). When the sound waves were applied to the sensor with polarized and interlocked P(VDF‐TrFE) layers, well‐synchronized time‐dependent voltage outputs were observed along with the corresponding spectrogram.

The rapid triboelectric response of the sensor developed in this study enables the spatial and temporal detection of surface textures. Figure 6d shows a schematic of texture perception by the sensor of fingerprint‐like polydimethylsiloxane (PDMS) line patterns for the amplification of the triboelectric output, which mimics the amplification of tactile signals by fingerprint patterns. Figure 6e shows the time‐dependent triboelectric output when the PDMS line pattern surface was scanned (0.5–2.5 mm s^−1^) by the sensor with a vertical force of 0.3 N for texture perception. With increasing scanning speed, the intensity and number of periodic spike peaks increased because of the regular topological features on the scanned surface. By converting these time‐dependent outputs into the frequency domain using fast Fourier transform (FFT) analysis, the fundamental frequencies (*f*) and its harmonics were obtained, which are correlated with the scanning speed (*v*) and grating period (*λ*) by *f = v/λ*, where the grating period of the line pattern is 500 µm (Figure 6f). Visualizing the STFT based on triboelectric outputs provides time‐dependent frequency information, which displays the intensity of the STFT magnitude over time (Figure 6g). The corresponding fundamental frequencies increased from 1 to 5 Hz as the amplitude increased when the sensor scanned over the line pattern surfaces at 0.5–2.5 mm s^−1^. Moreover, the sensor can detect the roughness of target objects. Figure 6h presents SEM images and the corresponding STFTs of sandpapers with different roughness values. Time‐dependent STFT analysis of the triboelectric current output revealed that the intensity of the STFT magnitude increased with increasing surface roughness.

Furthermore, the multimodality of the sensor allows the simultaneous application of pressure and temperature to be monitored in real time (Figure 6i–l). When the sensors were pressed by fingers with different temperatures (≈30 and ≈17 °C), the output signal from the warm finger consisted of the same directional triboelectric and pyroelectric peaks, while the output signal from the cold finger was composed of peaks with opposite directions (Figure 6i). For large‐scale multimodal sensing, we fabricated a 3 × 3 sensor array, as illustrated in Figure 6j. When objects (i–vi) with different weights and temperatures were placed on the sensor array, each spot detected and distinguished the simultaneously applied pressure and temperature based on the triboelectric and pyroelectric output signals (Figure 6k,l), which agreed with those obtained from the single device in Figure 5. These results demonstrate that inversely polarized P(VDF‐TrFE) with an interlocked microstructure leads to promising triboelectric and pyroelectric performances for various potential applications, such as self‐powered and multimodal sensors for healthcare, environmental monitoring, security, and humane–machine interfaces.

## 3 Conclusion

In summary, we demonstrated self‐powered multimodal sensors capable of distinguishing simultaneous pressure and temperature stimuli in a single device by profiling the coupled triboelectric and pyroelectric signals based on differences in the electron flow direction and response time. A remarkable multimodality was realized by introducing an interlocked microstructure of a ferroelectric copolymer (P(VDF‐TrFE)), which is attributed to two factors. One is the structural advantage for both triboelectric and pyroelectric performances owing to the micro‐gap and contact sites between the P(VDF‐TrFE) microridges. The other is the highly localized E‐field around the interlocked microstructures during ferroelectric polarization. The resultant sensor exhibits ultrasensitive pressure and temperature detection capability (2.2 V kPa^−1^, 0.27 nA °C^−1^) within a broad detection range (0.1–98 kPa, −20 °C < Δ*T* < 30 °C). Notably, our sensor provides the desired characteristics, such as facile fabrication, simple structure, discrimination of multiple stimuli without interference, and self‐powered ability, to fulfill practical demands for healthcare, human–machine interfaces, robotics, and artificial intelligence systems.

## 4 Experimental Section

### Synthesis of Microstructured P(VDF‐TrFE) Films and Fabrication of Devices

A P(VDF‐TrFE) solution (20 wt%) was prepared by dissolving 70/30 P(VDF‐TrFE) copolymer powder (Piezotech, France) in 99.5% *N*,*N*‐dimethylformamide (DMF). The P(VDF‐TrFE) solution was spin‐coated on a microstructured PDMS mold (1000 rpm, 60 s), followed by drying at 70 °C for 1 h to remove the DMF solvent. To enhance the crystallinity, the film was annealed at 140 °C for 2 h, followed by gradual natural cooling to room temperature. The as‐prepared film was transferred onto a Ni/Cu fabric electrode. Electrical poling was performed under an external E‐field (40 MV m^−1^) to align the electric dipoles. For the fabrication of free‐standing triboelectric devices (≈2 × 2 cm^2^), inversely polarized P(VDF‐TrFE) microstructured films were overlapped to face each other without an additional spacer.

### Characterization

The open‐circuit voltage and short‐circuit current were measured using an oscilloscope (DPO 2022 B, Tektronix, US) and a source meter (2450‐SCPI, Keithley, US), respectively. The output characteristics of the sensor were measured by applying a periodic force using a pushing tester (JIPT‐100, JUNIL TECH, Korea). The same condition of pusher movement (speed of 100 mm s^−1^, acceleration of 400 mm s^−2^, and delay of 1s after loading) was used for the application of force and temperature separately or simultaneously. As a thermal stimulus for pyroelectricity, a vial filled with glycerol was used after heating and cooling in advance. To apply the specific Δ*T* of thermal stimulus, the temperature of vial was monitored through IR image in real time during measurement. FEM simulations of the E‐field and polarization distribution in the diverse compositions of P(VDF‐TrFE) bilayers with planar and interlocked microstructures (diameter of 10 µm, pitch of 15 µm, and height of 6 µm) were performed using COMSOL Multiphysics software under Dirichlet boundary conditions (see details in Section S1, Supporting Information).

## Conflict of Interest

The authors declare no conflict of interest.

## Supporting information

Supporting Information

## Data Availability

The data that support the findings of this study are available from the corresponding author upon reasonable request.

## References

[1] T. Someya , M. Amagai , Nat. Biotechnol. 2019, 37, 382. 30940942 10.1038/s41587-019-0079-1

[2] Y. Lee , J. Park , A. Choe , S. Cho , J. Kim , H. Ko , Adv. Funct. Mater. 2020, 30, 1904523.

[3] H. R. Lim , H. S. Kim , R. Qazi , Y. T. Kwon , J. W. Jeong , W. H. Yeo , Adv. Mater. 2020, 32, 1901924. 10.1002/adma.201901924 31282063

[4] W. W. Lee , Y. J. Tan , H. Yao , S. Li , H. H. See , M. Hon , K. A. Ng , B. Xiong , J. S. Ho , B. C. K. Tee , Sci. Robot. 2019, 4, eaax2198. 33137772 10.1126/scirobotics.aax2198

[5] A. Chortos , J. Liu , Z. Bao , Nat. Mater. 2016, 15, 937. 27376685 10.1038/nmat4671

[6] Y. Gao , L. Yu , J. C. Yeo , C. T. Lim , Adv. Mater. 2020, 32, 1902133. 10.1002/adma.201902133 31339200

[7] J. Kim , A. S. Campbell , B. E.‐F. de Ávila , J. Wang , Nat. Biotechnol. 2019, 37, 389. 30804534 10.1038/s41587-019-0045-y PMC8183422

[8] Y. Lee , J. Myoung , S. Cho , J. Park , J. Kim , H. Lee , Y. Lee , S. Lee , C. Baig , H. Ko , ACS Nano 2020, 15, 1795. 33369402 10.1021/acsnano.0c09581

[9] S. Lee , S. Franklin , F. A. Hassani , T. Yokota , M. O. G. Nayeem , Y. Wang , R. Leib , G. Cheng , D. W. Franklin , T. Someya , Science 2020, 370, 966. 33214278 10.1126/science.abc9735

[10] X. Wang , Y. Zhang , X. Zhang , Z. Huo , X. Li , M. Que , Z. Peng , H. Wang , C. Pan , Adv. Mater. 2018, 30, 1706738. 10.1002/adma.201706738 29411908

[11] J. Park , M. Kim , Y. Lee , H. S. Lee , H. Ko , Sci. Adv. 2015, 1, e1500661. 26601303 10.1126/sciadv.1500661 PMC4646817

[12] F. Zhang , Y. Zang , D. Huang , C.‐a. Di , D. Zhu , Nat. Commun. 2015, 6, 8356. 26387591 10.1038/ncomms9356 PMC4595753

[13] N. T. Tien , S. Jeon , D. I. Kim , T. Q. Trung , M. Jang , B. U. Hwang , K. E. Byun , J. Bae , E. Lee , J. B. H. Tok , Adv. Mater. 2014, 26, 796. 24493054 10.1002/adma.201302869

[14] T. Q. Trung , S. Ramasundaram , B. U. Hwang , N. E. Lee , Adv. Mater. 2016, 28, 502. 26607674 10.1002/adma.201504441

[15] S. Jeon , S.‐C. Lim , T. Q. Trung , M. Jung , N.‐E. Lee , Proc. IEEE 2019, 107, 2065.

[16] S. Zhao , R. Zhu , Adv. Mater. 2017, 29, 1606151. 10.1002/adma.201606151 28195430

[17] P. Zhu , Y. Wang , Y. Wang , H. Mao , Q. Zhang , Y. Deng , Adv. Energy Mater. 2020, 10, 2070161.

[18] D. H. Ho , Q. Sun , S. Y. Kim , J. T. Han , D. H. Kim , J. H. Cho , Adv. Mater. 2016, 28, 2601. 26833961 10.1002/adma.201505739

[19] Q. Hua , J. Sun , H. Liu , R. Bao , R. Yu , J. Zhai , C. Pan , Z. L. Wang , Nat. Commun. 2018, 9, 244. 29339793 10.1038/s41467-017-02685-9 PMC5770430

[20] F. Li , Y. Liu , X. Shi , H. Li , C. Wang , Q. Zhang , R. Ma , J. Liang , Nano Lett. 2020, 20, 6176. 32662654 10.1021/acs.nanolett.0c02519

[21] S. Han , N. U. H. Alvi , L. Granlöf , H. Granberg , M. Berggren , S. Fabiano , X. Crispin , Adv. Sci. 2019, 6, 1802128. 10.1002/advs.201802128 PMC6468975 31016118

[22] I. You , D. G. Mackanic , N. Matsuhisa , J. Kang , J. Kwon , L. Beker , J. Mun , W. Suh , T. Y. Kim , J. B.‐H. Tok , Science 2020, 370, 961. 33214277 10.1126/science.aba5132

[23] M. Ma , Z. Zhang , Z. Zhao , Q. Liao , Z. Kang , F. Gao , X. Zhao , Y. Zhang , Nano Energy 2019, 66, 104105.

[24] Y.‐E. Shin , S.‐D. Sohn , H. Han , Y. Park , H.‐J. Shin , H. Ko , Nano Energy 2020, 72, 104671.

[25] J. Kim , J. H. Lee , H. Ryu , J. H. Lee , U. Khan , H. Kim , S. S. Kwak , S. W. Kim , Adv. Funct. Mater. 2017, 27, 1700702.

[26] W. Seung , H. J. Yoon , T. Y. Kim , H. Ryu , J. Kim , J. H. Lee , J. H. Lee , S. Kim , Y. K. Park , Y. J. Park , Adv. Energy Mater. 2017, 7, 1600988.

[27] M. Kim , D. Park , M. M. Alam , S. Lee , P. Park , J. Nah , ACS Nano 2019, 13, 4640. 30875188 10.1021/acsnano.9b00750

[28] M. Urbieta , M. Barbry , Y. Zhang , P. Koval , D. Sánchez‐Portal , N. Zabala , J. Aizpurua , ACS Nano 2018, 12, 585. 29298379 10.1021/acsnano.7b07401

[29] Y. Lee , J. Lee , T. K. Lee , J. Park , M. Ha , S. K. Kwak , H. Ko , ACS Appl. Mater. Interfaces 2015, 7, 26421. 26575302 10.1021/acsami.5b09947

[30] S. K. Srivastava , A. Gencoglu , A. R. Minerick , Anal. Bioanal. Chem. 2011, 399, 301. 20967429 10.1007/s00216-010-4222-6

[31] J. Park , J. Kim , J. Hong , H. Lee , Y. Lee , S. Cho , S.‐W. Kim , J. J. Kim , S. Y. Kim , H. Ko , NPG Asia Mater. 2018, 10, 163.

[32] C. V. Crowther , M. A. Hayes , Analyst 2017, 142, 1608. 28394391 10.1039/c6an02509a PMC5507384

[33] M. Saucedo‐Espinosa , B. Lapizco‐Encinas , J. Chromatogr. A 2015, 1422, 325. 26518498 10.1016/j.chroma.2015.10.030

[34] N. Bennaji , I. Mellouki , N. Yacoubi , Sens. Transducers 2014, 27, 75.

[35] I. Lubomirsky , O. Stafsudd , Rev. Sci. Instrum. 2012, 82, 121101. 10.1063/1.4709621 22667595

[36] C. Wang , X. Li , H. Hu , L. Zhang , Z. Huang , M. Lin , Z. Zhang , Z. Yin , B. Huang , H. Gong , Nat. Biomed. Eng. 2018, 2, 687. 30906648 10.1038/s41551-018-0287-x PMC6428206

[37] A. Kumar , R. Anel , E. Bunnell , K. Habet , S. Zanotti , S. Marshall , A. Neumann , A. Ali , M. Cheang , C. Kavinsky , Crit. Care Med. 2004, 32, 691. 15090949 10.1097/01.ccm.0000114996.68110.c9

[38] A. Al‐Qatatsheh , Y. Morsi , A. Zavabeti , A. Zolfagharian , N. Salim , A. Z. Kouzani , B. Mosadegh , S. Gharaie , Sensors 2020, 20, 4484. 10.3390/s20164484 PMC7474433 32796604

[39] W. W. Nichols , Am. J. Hypertens. 2005, 18, 3S. 15683725 10.1016/j.amjhyper.2004.10.009

[40] K. Kohara , Y. Tabara , A. Oshiumi , Y. Miyawaki , T. Kobayashi , T. Miki , Am. J. Hypertens. 2005, 18, 11S. 15683726 10.1016/j.amjhyper.2004.10.010

