# Supplementary material for: Ultrasensitive Multimodal Tactile Sensors with Skin‐Inspired Microstructures through Localized Ferroelectric Polarization
Source: Adv Sci (Weinh). 2022 Jan 24;9(9):2105423. doi: 10.1002/advs.202105423 (PMC8948547; doi:10.1002/advs.202105423)
Supplement: Supplementary file 1 — Supporting Information [file ADVS-9-2105423-s001.pdf]

ADVANCED SCIENCE
Open Access

Supporting Information

for *Adv. Sci.*, DOI 10.1002/advs.202105423

Ultrasensitive Multimodal Tactile Sensors with Skin-Inspired Microstructures through Localized Ferroelectric Polarization

*Young-Eun Shin, Yong-Jin Park, Sujoy Kumar Ghosh, Youngoh Lee, Jonghwa Park and Hyunhyub Ko**

ADVANCED SCIENCE
Open Access

Supporting Information

for *Adv. Sci.*, DOI: 10.1002/advs.202105423

Ultrasensitive Multimodal Tactile Sensors with Skin-Inspired Microstructures through Localized Ferroelectric Polarization

*Young-Eun Shin, Yong-Jin Park, Sujoy Kumar Ghosh, Youngoh Lee, Jonghwa Park, Hyunhyub Ko**

Supporting Information

Ultrasensitive Multimodal Tactile Sensors with Skin-Inspired Microstructures through Localized Ferroelectric Polarization

*Young-Eun Shin, Yong-Jin Park, Sujoy Kumar Ghosh, Youngoh Lee, Jonghwa Park, Hyunhyub Ko**

Note S1. Device simulations

Electric field simulation

The finite element method (FEM) simulations were performed by solving Maxwell's equations. To model the electric field within the dielectric under static conditions, a scalar potential was used (Equation (S1)) with Dirichlet boundary conditions (Equation (S2)).

$$-\nabla.\left[\frac{1}{\rho(x,y)}\nabla V(x,y)\right]=0 \quad \text{(S1)}$$

$$\mathrm{V}(\mathrm{x},\mathrm{y})=\upsilon_0 \text{ on } \Gamma_0,\ \mathrm{V}(\mathrm{x},\mathrm{y})=\upsilon_1 \text{ on } \Gamma_1 \quad \text{(S2)}$$

Here, υ_0 and υ_1 are the voltages applied to the electrodes bounded by Γ_0 and Γ_1, respectively. In Equation (S1) and (S2), $V(x,y)$ represents the scalar potential, and $\rho(x,y)$ is the space charge density (assuming free space). The electric field is expressed in terms of the scalar potential: $\vec{E}(x,y) = -\nabla V(x,y)$.

In this case, Γ_0 represents the boundary between the bottom electrode and dielectric, and Γ_1 represents the boundary between the top electrode and dielectric. The values of υ_0 and υ_1 were set to 0 and 1400 V, respectively.

Because a high voltage is applied in this case, space charges are not present or are negligible (i.e., $\rho(x,y)=0$). Therefore, the resulting equations are Laplace's equations: $\nabla V^2(x,y)=0$. In the field of electrostatics, the electrical conductivity(σ) is zero for an ideal insulator and infinite for an ideal conductor.

A capacitor structure was considered for the electric-field simulation, as shown in Figure 1. The capacitor was placed in the air domain because there can be significant fringing fields around the capacitor plates. The size of the air volume truncates the modeling space. While the fringing

electric fields extend to infinity, they drop off proportional to the inverse cube of the distance. Consequently, they rapidly become sufficiently small to be considered numerically insignificant. Here, it is assumed that the air volume is sufficiently large to accurately capture the fringing fields.

Polarization simulation

An idealized dielectric material is characterized by its lack of free charges; instead, it has bound charges. At the microscopic level, these bound charges can be displaced by an external electric field, resulting in induced electric dipoles. These induced electric dipoles are pairs of positive and negative charges that align with the electric field. This results in an electric field inside a dielectric material that differs from that of free space. To obtain a macroscopic description of this phenomenon, it is convenient to introduce a polarization vector field ($\vec{P}$) and polarization charge density (ρ_p). They are related by $\rho_p = -\nabla.\vec{P}$.

The polarization effects locally modify the electric field inside a material according to $\nabla.\vec{E} = \frac{\rho+\rho_p}{\epsilon_0}$, or equivalently, $\nabla.(\epsilon_0\vec{E} + \vec{P}) = \rho$.

Based on this, a new fundamental quantity can be introduced: the electric displacement field ($\vec{D}$), which is defined as $\vec{D} = \epsilon_0\vec{E} + \vec{P}$. Using this definition, the electrostatic equation, also known as Gauss's law, becomes $\nabla.\vec{D} = \rho$.

To fully describe electrostatic phenomena, the condition that the electric field is irrotational (Faraday's law) must be enforced. Because this condition is encoded in the electric potential, the equations of electrostatics can be combined into a single equation: $-\nabla.(\epsilon_0\nabla V - \vec{P}) = \rho$.

By solving this equation, the polarization field was evaluated in the FEM simulations using the same boundary conditions as those described above.

The overall simulation was performed using the "electrostatics (es)" COMSOL module.

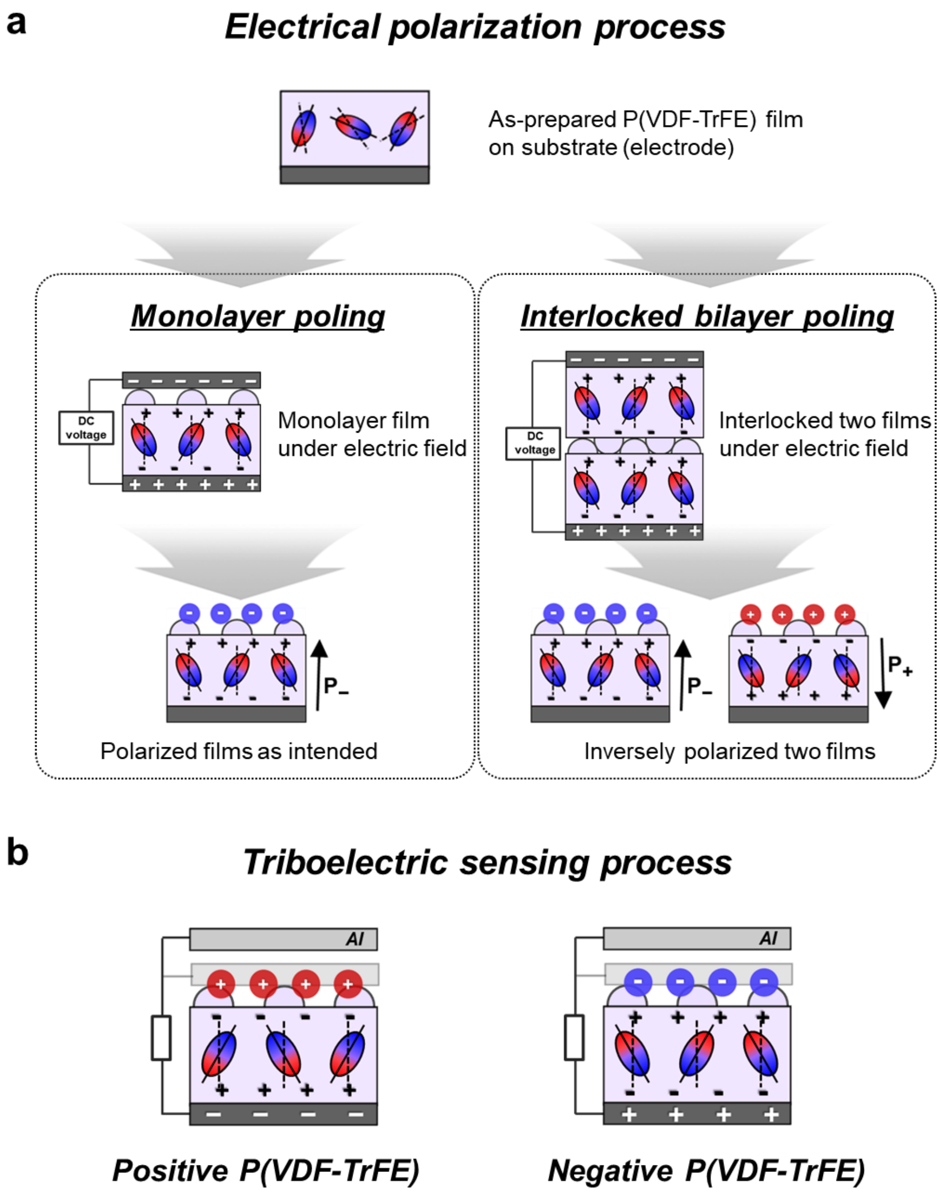

Figure S1. (a) Schematic of the electrical polarization process for monolayer poling and interlocked bilayer poling. (b) Schematic of the triboelectric sensing process with positive and negative Poly(vinylidene fluoride-trifluoroethylene) (P(VDF-TrFE)) layers.

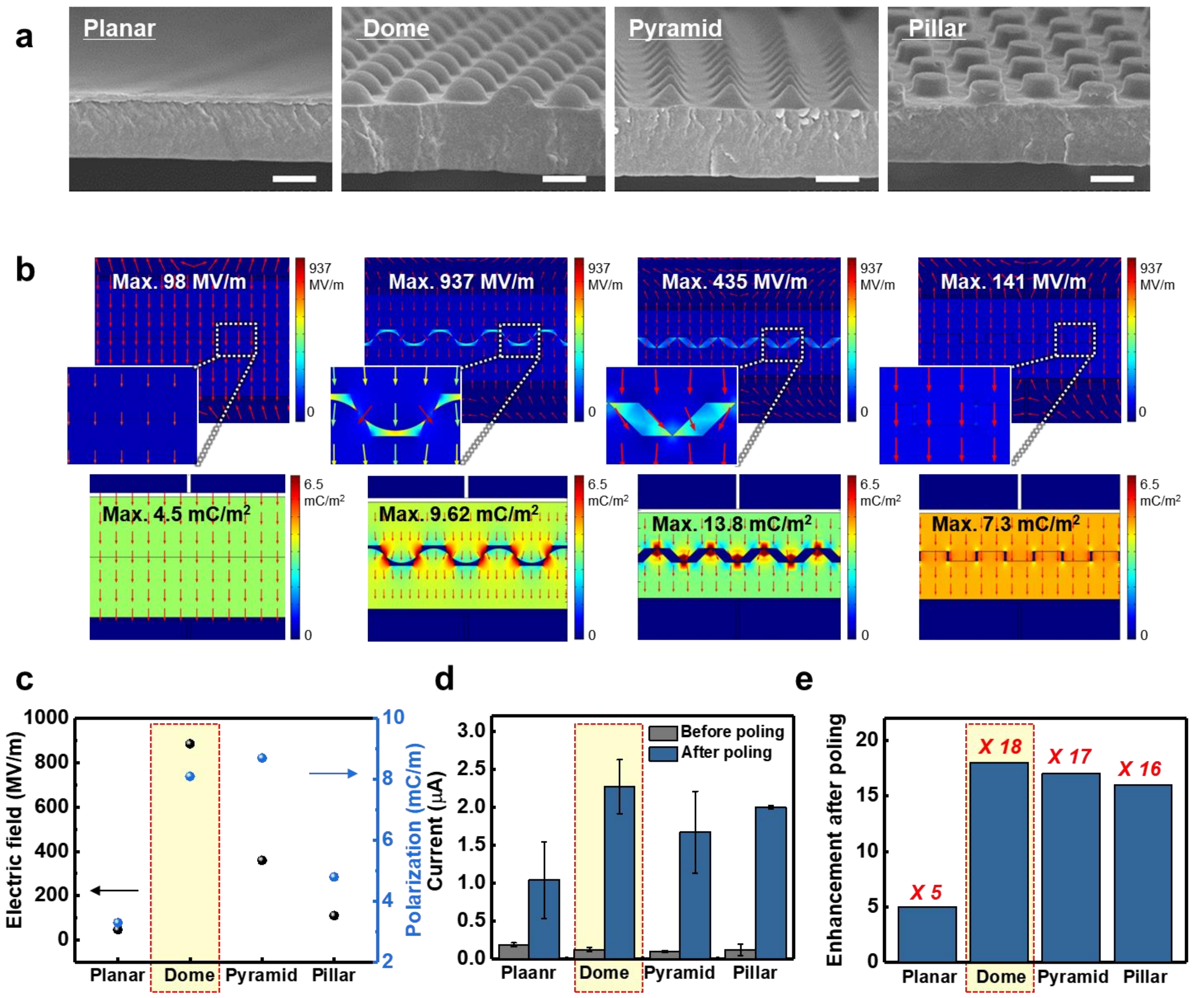

Figure S2. Dependence of localized ferroelectric polarization and triboelectric performance on microstructure shape. (a) Scanning electron microscopy (SEM) images of different microstructure morphologies (scale bar is 10 μm): planar, microdome, micropyramid, and micropillar. (b) Contours plots and (c) comparison of simulated electric-field distribution and polarization for differently shaped microstructures. (d) Triboelectric output current and (e) enhancement after poling for differently shaped microstructures.

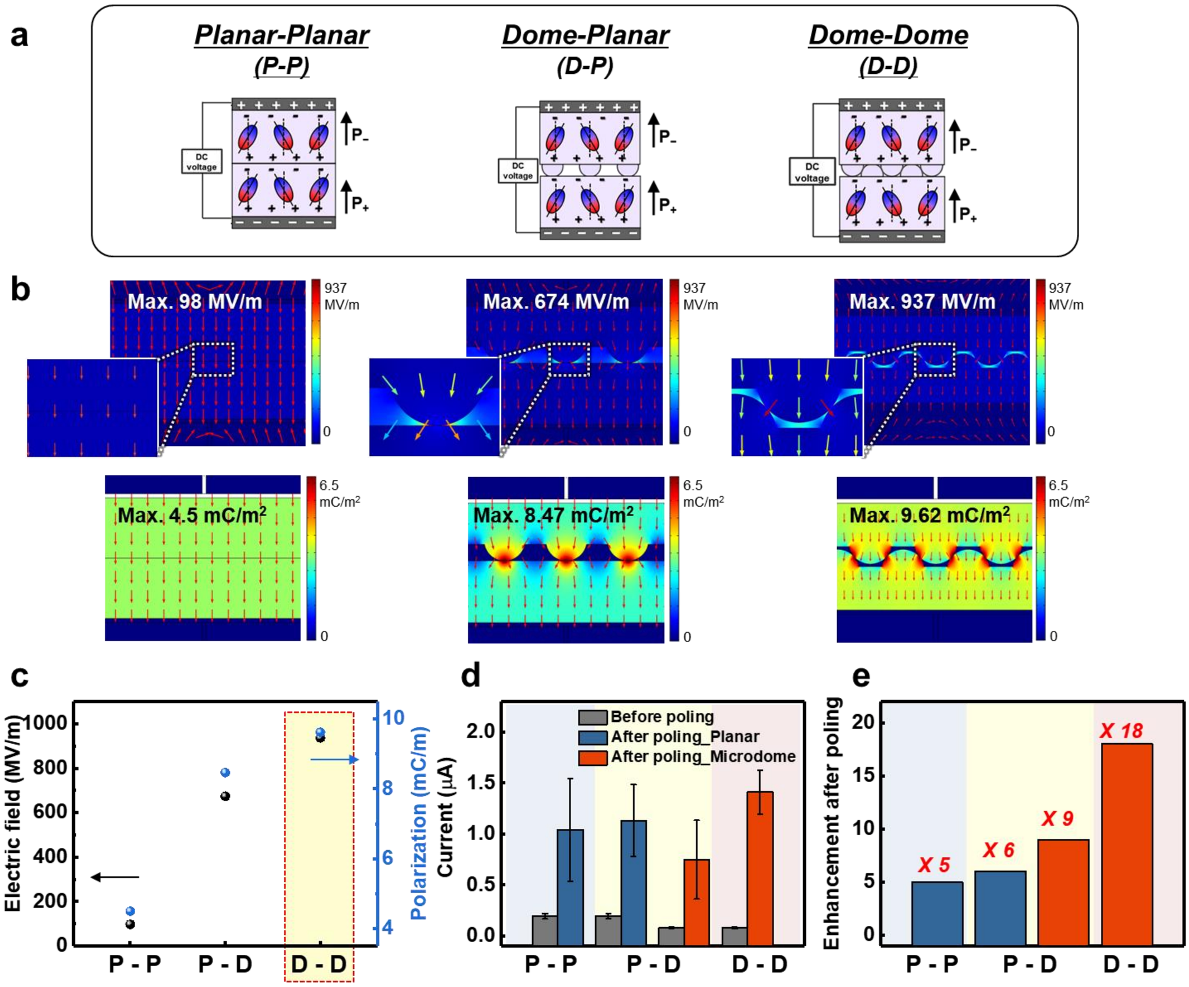

Figure S3. Dependence of localized ferroelectric polarization and triboelectric performance on device composition. (a) Schematic of different device compositions. (b) Contours plots and (c) comparison of simulated electric-field distribution and polarization for different device compositions. (d) Triboelectric output current and (e) enhancement after poling for different device compositions.

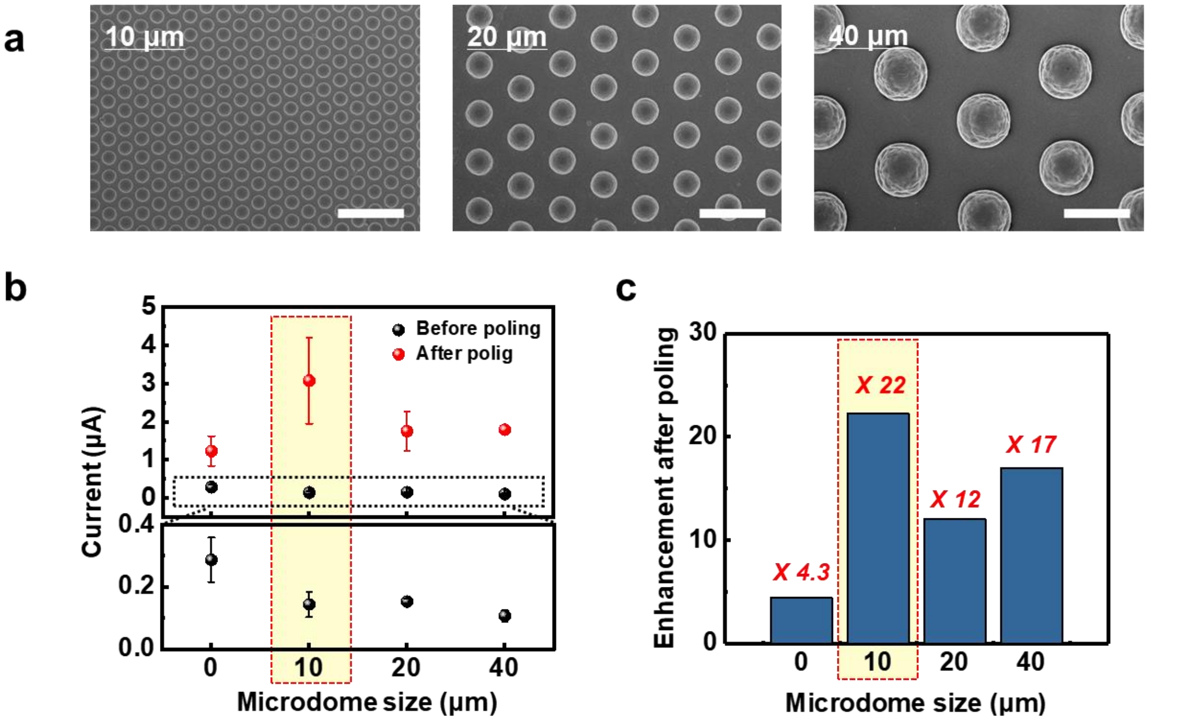

Figure S4. Dependence of triboelectric performance on microdome diameter. (a) SEM images of microdome structures with different diameters (scale bar is 50 μm): 10, 20, and 40 μm. (b) Triboelectric output current and (c) enhancement after poling for different microdome sizes.

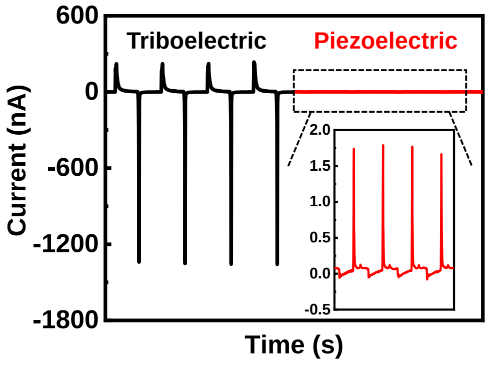

Figure S5. Comparison of triboelectric and piezoelectric output performances from planar structure of as-poled P(VDF-TrFE) films under the same pressure with 98kPa.

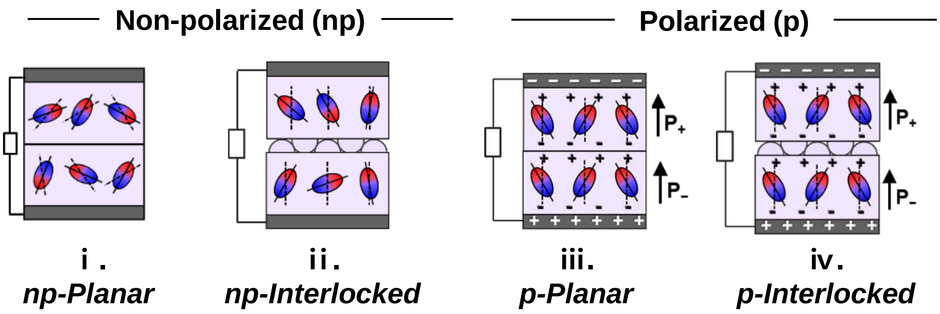

Figure S6. Schematic of different types of sensors for pressure-sensitive triboelectric performance: (i) non-polarized (np)-planar, (ii) np-interlocked, (iii) polarized (p)-planar, and (iv) p-interlocked.

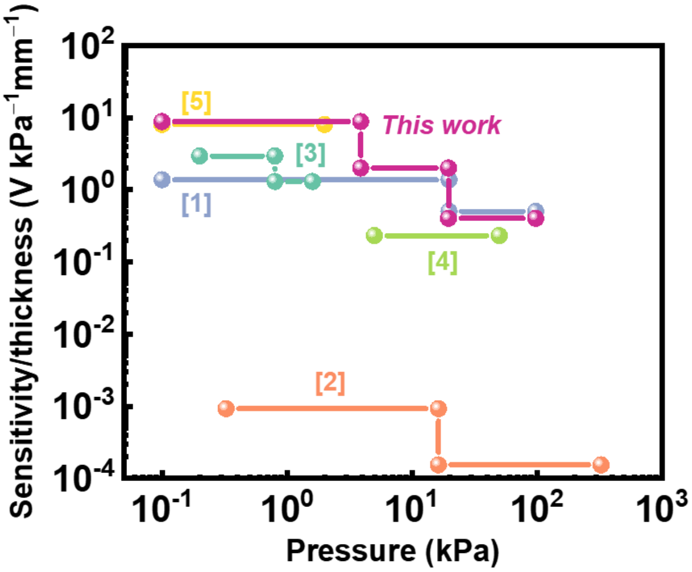

Figure S7. Comparison of pressure sensitivity considering device thickness and detection range of microstructure pressure sensor.[1-5]

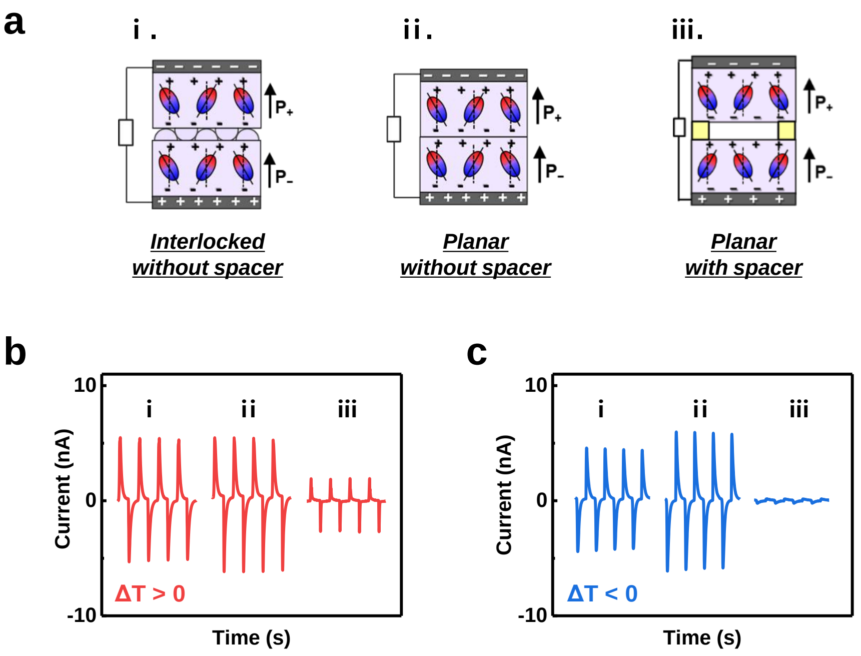

Figure S8. Comparison of pyroelectric performances of different types of devices. (a) Schematic of different types of devices: (i) interlocked structure without a spacer, (ii) planar structure without a spacer, and (iii) planar structure with a spacer. (b, c) Pyroelectric output current of different types of devices under (b) $\Delta T > 0$ and (c) $\Delta T < 0$.

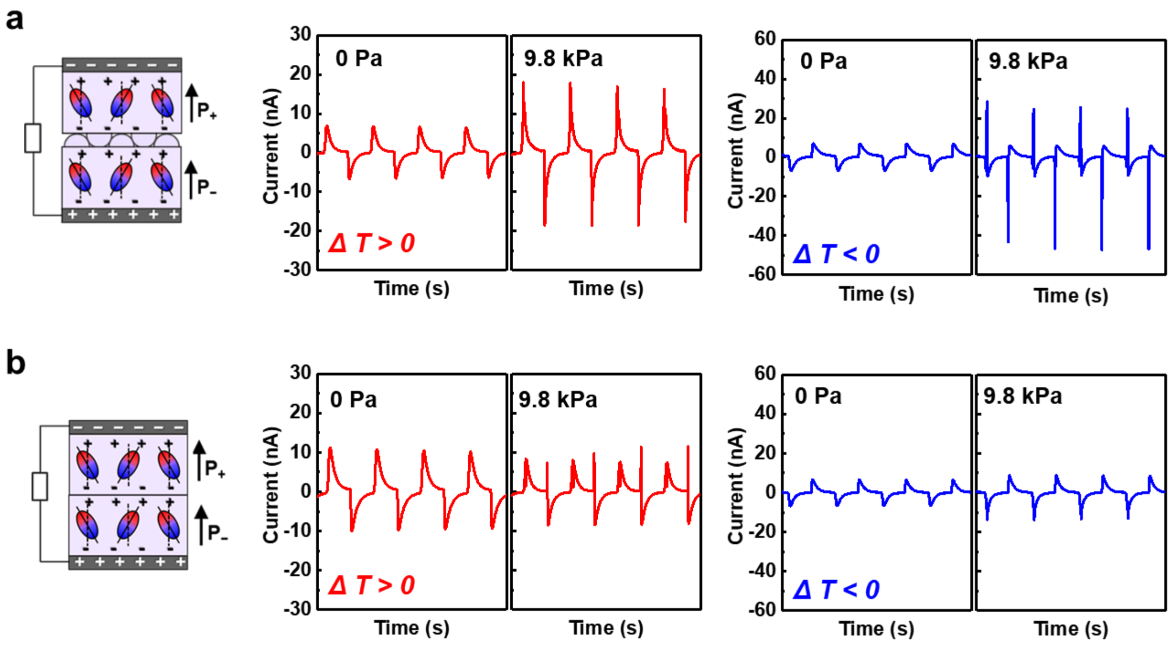

Figure S9. Comparison of multimodality between interlocked and planar microstructures. Multimodal signals under simultaneous pressure and temperature from (a) interlocked and (b) planar microstructures.

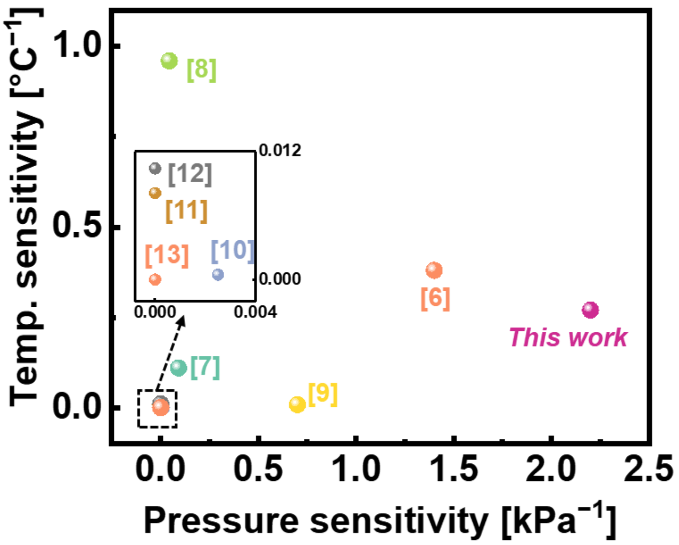

Figure S10. Comparison of multimodal sensors according to their pressure and temperature sensitivities.[6-13]

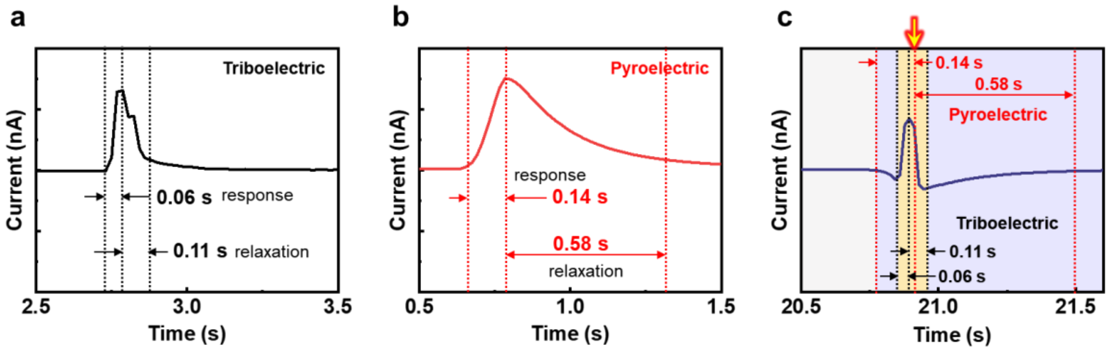

Figure S11. Deconvolution of multimodal signal on the basis of response and relaxation time from triboelectriccity and piezoelectricity. (a) Triboelectric signal under 1.96 kPa, (b) pyroelectric signal under ΔT 20 °C, and (c) multimodal signals under 9.8 kPa and ΔT −10 °C; yellow arrow points the expected position of the pyroelectric peak overlapped in triboelectric peak.

Table S1. Comparison of pressure sensing performances of microstructure sensor considering device thickness.

Ref.	Major materials	Range	Sensitivity	Device thickness	Sensitivity/thickness
This work	Interlocked PVDF-TrFE microstructure	0.1 – 98 kPa	2.2 V kPa^{-1} (0.1 – 3.9 kPa) 0.5 V kPa^{-1} (3.9 – 19.6 kPa) 0.1 V kPa^{-1} (19.6 – 98 kPa)	~250 μm (~50 μm except for electrode)	8.8 V $kPa^{-1}mm^{-1}$
[1]	Porous PVDF & PDMS	< 100 kPa	0.55 V kPa^{-1} (< 19.8 kPa) 0.2 V kPa^{-1} (19.8 – 100 kPa)	~400 μm	1.38 V $kPa^{-1}mm^{-1}$
[2]	PVDF fiber & Nylon fabric	0.326 – 326 kPa	6.23 mV kPa^{-1} (326 Pa – 16.3 kPa) 1.12 mV kPa^{-1} (16.3 ~ 326 kPa)	~6.5 mm	0.00096 V $kPa^{-1}mm^{-1}$
[3]	Electrospun PVP & PVDF fiber	0.2 – 2 kPa	8.8 V kPa^{-1} (200 – 800 Pa) 3.9 V kPa^{-1} (800 – 1400 Pa)	~3 mm (except for electrode)	2.93 V $kPa^{-1}mm^{-1}$
[4]	Microstructured PDMS & PTFE tiny burrs	5 – 50 kPa	127.22 mV kPa^{-1}	> 550 μm (except for shielding film & back electrode)	0.23 V $kPa^{-1}mm^{-1}$
[5]	Hierarchically Microstructured PDMS	0.1 – 60 kPa	7.989 V kPa^{-1} (0.1 – 2 kPa)	> 1 mm	7.89 V $kPa^{-1}mm^{-1}$

Table S2. Comparison of multimodal sensing performance according to pressure and temperature detection.

Ref.	Type	Pressure sensitivity	Temeprature sensitivity	Zero-bias
This work	Triboelectric/ Pyroelectric	(0.1 – 3.9 kPa)<br>2.2 V kPa^{-1}	(−20 ℃ < ΔT < 30 ℃)<br>0.16 nA $℃^{-1}$ @ $\Delta T > 0$<br>0.27 nA $℃^{-1}$ @ $\Delta T < 0$<br>Response time 0.15s	○
[6]	Triboelectric, Pyroelectric	(0.098 – 19.6 kPa)<br>40 nA kPa^{-1} ,<br>1.4 V kPa^{-1}	(−20 ℃ < ΔT < 20 ℃)<br>0.27 nA $℃^{-1}$ @ $\Delta T > 0$<br>0.38 nA $℃^{-1}$ @ $\Delta T < 0$<br>Response time 0.16s	○
[7]	Triboelectric, Piezoelectric, Pyroelectric	(10.5 – 52 kPa)<br>0.092 V kPa^{-1}	(0 ℃ < T < 58 ℃)<br>0.11 V $℃^{-1}$<br>Response time 0.53 s	○
[8]	Pyroelectric, Piezoelectric	(15.4 – 27.6 kPa)<br>0.044 V kPa^{-1}	(2.03℃ < ΔT < 13.57 ℃)<br>0.96 nA $℃^{-1}$, 0.048 V $℃^{-1}$	○
[9]	Capacitive, NTC thermistor	(0 – 25 kPa)<br>0.7 kPa^{-1}	(22 ℃ < T < 70 ℃)<br>0.83 % K^{-1}	X
[10]	Piezo-resistive, NTC thermistor	(0.1 – 102 kPa)<br>0.25 % kPa^{-1}	(25 ℃ < T < 100 ℃)<br>4.8 E^{-4} $℃^{-1}$	X
[11]	Resistive, Thermoelectric (electronic/ionic thermovoltaic)	-	(0 K < T < 20 K)<br>8100 μV K^{-1}	X
[12]	Resistive, Capacitive	-	10.4% $℃^{-1}$	X
[13]	Resistive, Thermoelectric	-	(0 ℃ < T < 9 ℃)<br>− 25.3 μV K^{-1}	X

References

[1] M. Ha, S. Lim, S. Cho, Y. Lee, S. Na, C. Baig, H. Ko, *ACS Nano* **2018**, 12, 3964.

[2] Y.-E. Shin, J.-E. Lee, Y. Park, S.-H. Hwang, H. G. Chae, H. Ko, *J. Mater. Chem. A* **2018**, 6, 22879.

[3] C. Garcia, I. Trendafilova, R. G. de Villoria, J. S. del Rio, *Nano Energy* **2018**, 50, 401.

[4] G. Yao, L. Xu, X. Cheng, Y. Li, X. Huang, W. Guo, S. Liu, Z. L. Wang, H. Wu, *Adv. Funct. Mater.* **2020**, 30, 1907312.

[5] S. Chen, N. Wu, S. Lin, J. Duan, Z. Xu, Y. Pan, H. Zhang, Z. Xu, L. Huang, B. Hu, *Nano Energy* **2020**, 70, 104460.

[6] Y.-E. Shin, S.-D. Sohn, H. Han, Y. Park, H.-J. Shin, H. Ko, *Nano Energy* **2020**, 72, 104671.

[7] M. Ma, Z. Zhang, Z. Zhao, Q. Liao, Z. Kang, F. Gao, X. Zhao, Y. Zhang, *Nano Energy* **2019**, 66, 104105.

[8] K. Song, R. Zhao, Z. L. Wang, Y. Yang, *Adv. Mater.* **2019**, 31, 1902831.

[9] G. Y. Bae, J. T. Han, G. Lee, S. Lee, S. W. Kim, S. Park, J. Kwon, S. Jung, K. Cho, *Adv. Mater.* **2018**, 30, 1803388.

[10] S. Chun, W. Son, C. Choi, H. Min, J. Kim, H. J. Lee, D. Kim, C. Kim, J.-s. Koh, C. Pang, *ACS Appl. Mater. Interfaces* **2019**, 11, 13608.

[11] S. Han, N. U. H. Alvi, L. Granlöf, H. Granberg, M. Berggren, S. Fabiano, X. Crispin, *Adv. Sci.* **2019**, 6, 1802128.

[12] I. You, D. G. Mackanic, N. Matsuhisa, J. Kang, J. Kwon, L. Beker, J. Mun, W. Suh, T. Y. Kim, J. B.-H. Tok, *Science* **2020**, 370, 961.

[13] F. Li, Y. Liu, X. Shi, H. Li, C. Wang, Q. Zhang, R. Ma, J. Liang, *Nano Lett.* **2020**, 20, 6176.
